# A Novel Class of Plant Type III Polyketide Synthase Involved in Orsellinic Acid Biosynthesis from *Rhododendron dauricum*

**DOI:** 10.3389/fpls.2016.01452

**Published:** 2016-09-27

**Authors:** Futoshi Taura, Miu Iijima, Eriko Yamanaka, Hironobu Takahashi, Hiromichi Kenmoku, Haruna Saeki, Satoshi Morimoto, Yoshinori Asakawa, Fumiya Kurosaki, Hiroyuki Morita

**Affiliations:** ^1^Graduate School of Medicine and Pharmaceutical Sciences for Research, University of ToyamaToyama, Japan; ^2^Graduate School of Pharmaceutical Sciences, Kyushu UniversityFukuoka, Japan; ^3^Institute of Pharmacognosy, Tokushima Bunri UniversityTokushima, Japan; ^4^Institute of Natural Medicine, University of ToyamaToyama, Japan

**Keywords:** biosynthesis, plant type III polyketide synthase, orcinol, orsellinic acid, daurichromenic acid, *Rhododendron dauricum*

## Abstract

*Rhododendron dauricum* L. produces daurichromenic acid, the anti-HIV meroterpenoid consisting of sesquiterpene and orsellinic acid (OSA) moieties. To characterize the enzyme responsible for OSA biosynthesis, a cDNA encoding a novel polyketide synthase (PKS), orcinol synthase (ORS), was cloned from young leaves of *R. dauricum*. The primary structure of ORS shared relatively low identities to those of PKSs from other plants, and the active site of ORS had a unique amino acid composition. The bacterially expressed, recombinant ORS accepted acetyl-CoA as the preferable starter substrate, and produced orcinol as the major reaction product, along with four minor products including OSA. The ORS identified in this study is the first plant PKS that generates acetate-derived aromatic tetraketides, such as orcinol and OSA. Interestingly, OSA production was clearly enhanced in the presence of *Cannabis sativa* olivetolic acid cyclase, suggesting that the ORS is involved in OSA biosynthesis together with an unidentified cyclase in *R. dauricum*.

## Introduction

**FIGURE 1:**
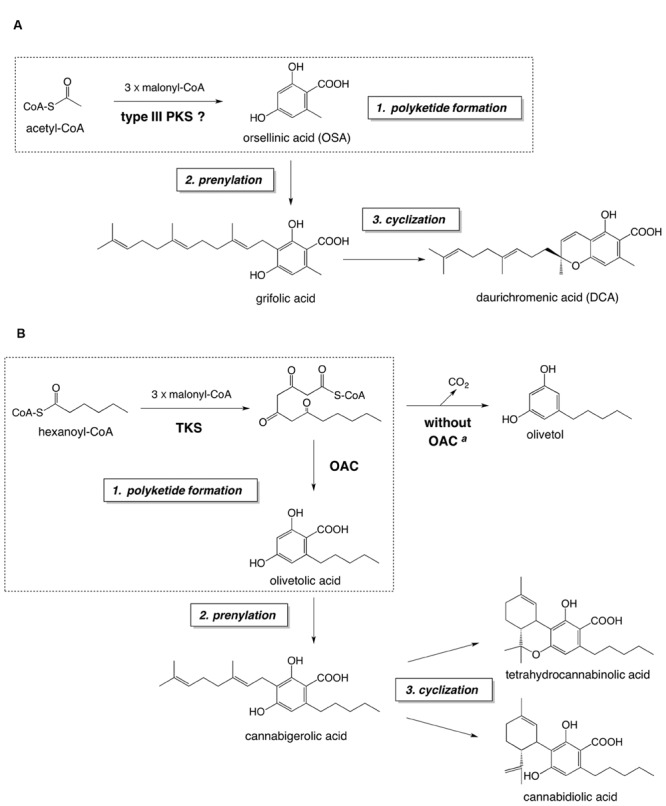
**The proposed biosynthetic pathway of DCA **(A)** and the established biosynthetic route to major cannabinoids **(B)**.** Both pathways consist of (1) polyketide formation, (2) prenylation, and (3) cyclization steps. The polyketide formation steps of each pathway are enclosed in dashed squares. ^a^In the absence of OAC, the pentyl tetra-β-ketide intermediate is spontaneously cyclized to olivetol via decarboxylative aldol condensation. Abbreviations used are: OAC, olivetolic acid cyclase; TKS, tetraketide synthase.

*Rhododendron dauricum* L. (Ericaceae) produces daurichromenic acid (DCA), the unique meroterpenoid composed of sesquiterpene and orsellinic acid (OSA) moieties (Kashiwada et al., 2001) (**Figure 1A**). DCA has attracted considerable attention because it displays various interesting pharmacological activities, including a potent anti-HIV effect (Iwata et al., 2004; Hashimoto et al., 2005; Tokuyama et al., 2007; Iwata and Kitanaka, 2010; Lee, 2010). Thus, chemical syntheses of DCA have been extensively studied over the past few years (Liu and Woggon, 2010; Bukhari et al., 2015; Luo et al., 2015; Elliott et al., 2016).

We presumed that DCA is biosynthesized via a pathway similar to that for cannabinoid biosynthesis in *Cannabis sativa* L. (Taura et al., 2007a; Vickery et al., 2016), because of the structural similarity between DCA and cannabinoids (**Figure 1**). The proposed DCA pathway and the cannabinoid biosynthesis consist of three reaction steps: (1) polyketide formation, (2) prenylation, and (3) cyclization, to synthesize cyclic meroterpenoids as final metabolites (**Figure 1**). Previously, we identified a DCA synthase in the young leaves of *R. dauricum*, which catalyzes the “cyclization step” to form DCA from grifolic acid (Taura et al., 2014). Interestingly, the biochemical properties of DCA synthase were quite similar to those reported for cannabinoid synthases (Sirikantaramas et al., 2004; Taura et al., 2007b). However, in contrast to the cannabinoid pathway, in which all of the biosynthetic enzymes have been identified (Taura et al., 2007a; Vickery et al., 2016), the biosynthetic mechanism leading to grifolic acid in the DCA pathway has remained elusive. In this study, we focused on the polyketide synthase (PKS) involved in the OSA biosynthesis in *R. dauricum*.

OSA is the simplest alkylresorcylic acid, and is regarded as a polyketide synthesized from acetyl-CoA and three molecules of malonyl-CoA (Dewick, 2002). Actually, several iterative type I PKSs have been identified in fungi as OSA synthases (Weitnauer et al., 2001; Ahlert et al., 2002; Sanchez et al., 2010; Jørgensen et al., 2014; Li et al., 2016). However, OSA has not been reported as a plant type III PKS product, although various kinds of PKSs have been found in the plant kingdom (Austin and Noel, 2003; Abe and Morita, 2010). The type III PKSs are structurally simple homodimeric enzymes composed of 40–45 kDa subunits that share considerable sequence homology with each other. These enzymes utilize the conserved Cys-His-Asn catalytic triad to perform the sequential condensations of C2 units derived from malonyl-CoA to a starter-CoA molecule, and cyclize the linear intermediate in most cases (Austin and Noel, 2003; Abe and Morita, 2010). Structure-function analyses of plant PKSs have demonstrated that diverse PKSs have evolved from chalcone synthase (CHS), the ubiquitous plant type III PKS, by only a small number of amino acid substitutions (Ferrer et al., 1999; Jez et al., 2000; Austin et al., 2004). We thus expected that the *R. dauricum* PKS, involved in the OSA biosynthesis, might also be derived from CHS to open a metabolic entrance into the DCA pathway.

Hitherto, alkylresorcylic acid-producing plant type III PKSs have been cloned from *Oryza sativa*, based on genome mining studies (Matsuzawa et al., 2010). The recombinant *O. sativa* alkylresorcylic acid synthases (named ARAS1 and ARAS2) produced alkylresorcylic acids from starter-CoAs with medium to long alkyl side chains (C12 to C22). The substrate preference of the ARASs is similar to that of the 2′-oxoalkylresorcylic acid synthase from the filamentous fungi *Neurospora crassa*, which synthesizes pentaketide alkylresorcylic acids as major reaction products (Funa et al., 2007). Interestingly, acetyl-CoA, the precursor of OSA, is not a substrate for these type III PKSs from *O. sativa* and *N. crassa* (Funa et al., 2007; Matsuzawa et al., 2010). Recently, a unique biosynthetic mechanism has been reported for the formation of olivetolic acid, the first committed intermediate in the cannabinoid pathway in *C. sativa*, as shown in **Figure 1B**. With this scheme, olivetolic acid is biosynthesized by the co-actions of a type III PKS tetraketide synthase (TKS; Taura et al., 2009) and olivetolic acid cyclase (OAC; Gagne et al., 2012; Yang et al., 2016): OAC produces olivetolic acid via the C2–C7 aldol condensation of the pentyl tetra-β-ketide CoA, synthesized by TKS from hexanoyl-CoA and malonyl-CoA. When the TKS reaction is performed without OAC, olivetol was detected as the predominant product, instead of olivetolic acid (Taura et al., 2009), suggesting that the tetraketide produced by TKS tends to be spontaneously cyclized into olivetol via decarboxylative aldol condensation (**Figure 1B**). Thus, TKS was originally called olivetol synthase (Taura et al., 2009), until the partner enzyme OAC was discovered as the first plant polyketide cyclase (Gagne et al., 2012). As for the structural features, OAC is a dimeric α+β barrel type polyketide cyclase composed of 101-amino acid subunits, with a molecular mass of ∼12 kDa (Yang et al., 2016).

In this study, we cloned two PKS cDNAs from *R. dauricum*. One is a CHS cDNA, and the other encodes a novel PKS named orcinol synthase (ORS), as this enzyme produced orcinol as the major reaction product. The catalytic properties of ORS were characterized in the absence and presence of *C. sativa* OAC, to illustrate the reaction mechanism and the possible physiological function of this novel plant type III PKS.

## Materials and Methods

### Plant Material and Reagents

*Rhododendron dauricum* plants were cultivated in the Experimental Station for Medicinal Plant Research, at the University of Toyama. *p*-Coumaroyl-CoA was chemically synthesized (Abe et al., 2000). Other acyl-CoA esters were purchased from Sigma. Orcinol and OSA were obtained from Wako Pure Chemicals (Osaka, Japan). Triacetic acid lactone and phloroacetophenone were from Tokyo Chemical Industry Co., Ltd. (Tokyo, Japan). Tetraacetic acid lactone was purified from a *Pichia pastoris* culture expressing ORS, as described below. Other chemical reagents were purchased from Wako Pure Chemicals, and molecular biology reagents were from Takara Bio (Shiga, Japan), unless otherwise stated.

### RNA Extraction and Reverse Transcription

Total RNA was extracted from young leaves of *R. dauricum*, using an RNAqueous kit (Thermo Fisher Scientific). The first strand cDNA was synthesized from 1.0 μg of RNA, using the primer dT_17_AP (Supplementary Table 1) and ReverTra Ace (Toyobo, Osaka, Japan), according to manufacturer’s instructions. The template for 5′-rapid amplification of cDNA end (5′-RACE) was prepared by polyadenylation of the cDNA, using terminal deoxynucleotidyl transferase in the presence of deoxyadenine.

### Cloning and Sequencing of cDNAs Encoding *R. dauricum* ORS and CHS

The oligonucleotide primers and PCR conditions used in this study are listed in Supplementary Table 1. All cDNA fragments were amplified by using *ExTaq* DNA polymerase (Takara Bio). First, the core fragments for ORS and CHS (∼250 bp) were obtained by PCR, with the degenerate primers PKS_Fw and PKS_Rv. The 3′-terminal and 5′-terminal regions of the ORS cDNA were amplified by 3′- and 5′-RACE (Frohman et al., 1998). The 3′-RACE product (∼1,200 bp) was obtained by PCR with the gene-specific primer ORS_3R and the adapter primer AP. The 5′-RACE product (∼300 bp) was amplified as follows. The first round of PCR was performed with the gene-specific primer ORS_5R1 and the adapter primer dT_17_AP, in the presence of a poly(dA)-tailed cDNA. The cDNA fragment was obtained by nested PCR with the gene-specific primer ORS_5R2 and the adapter primer AP. Similar procedures were used for 3′-RACE and 5′-RACE for the CHS cDNA, except that the primers CHS_3R, CHS_5R1, and CHS_5R2 were used instead of ORS_3R, ORS_5R1, and ORS_5R2, respectively, to obtain the ∼1,000 bp 3′-RACE and ∼250 bp 5′-RACE products. All PCR products were cloned into the T-vector pMD19 (Takara Bio) and sequenced using a 3130 Genetic Analyzer (Thermo Fisher Scientific). The cDNA fragment sequences were assembled by BioEdit version 7.2.5^1^. The nucleotide sequence data of *R. dauricum* ORS and CHS were deposited in the DDBJ/EMBL/GenBank databases, under the accession numbers LC133082 and LC133083, respectively.

### Computational Analyses of ORS and CHS

The multiple alignment of the type III PKSs, including *R. dauricum* ORS and CHS, was made by the Clustal W program (Thompson et al., 1994). A neighbor-joining phylogenetic tree was drawn by 1,000 bootstrap tests with a *p*-distance matrix, using the MEGA6.06 software (Tamura et al., 2013). The homology model of ORS was generated with the SWISS-MODEL software (Schwede et al., 2003), using the crystal structure of *Medicago sativa* CHS2 (PDB ID: 1BI5) as the template. The model quality was evaluated by a Ramachandran plot, using RAMPAGE (Lovell et al., 2003). The cavity volume of the model was calculated with CASTp (Dundas et al., 2006). All protein figures were rendered with PyMOL^2^.

### Bacterial Expression and Purification of the Recombinant ORS and CHS

Full-length cDNAs were amplified with the gene-specific primers ORS_Fw and ORS_Rv (for ORS), or CHS_Fw and CHS_Rv (for CHS), using a proofreading polymerase (PrimeStar HS, Takara Bio). The amplified cDNAs were gel-purified and subcloned into the pQE80L vector (Qiagen), predigested with *Bam*HI and *Sal*I, using the In-Fusion HD cloning reagent (Takara Bio). The resulting constructs, which direct the synthesis of the recombinant proteins with an N-terminal hexahistidine tag, were individually transformed into *Escherichia coli* M15 (Qiagen).

*Escherichia coli* M15 cells harboring the recombinant pQE-80L plasmids were cultured in liquid LB medium, containing 25 μg/ml kanamycin and 100 μg/ml ampicillin. When the optical density of the culture at 600 nm reached 0.6, isopropyl-β-D-thiogalactoside (0.5 mM) was added to the culture, to induce the recombinant protein expression. After an incubation at 25°C for 5 h, the cells were harvested by centrifugation, resuspended in 50 ml of buffer A [20 mM Tris-HCl (pH 7.5) containing 0.1 M NaCl and 1 mM β-mercaptoethanol], and disrupted by sonication. The homogenate was centrifuged at 20,000 × *g* for 20 min, to remove insoluble materials. The supernatant was applied to a column (1.0 cm × 2.5 cm) containing Ni Sepharose 6 Fast Flow resin (GE Healthcare), equilibrated with buffer A. After the sample was applied to the column, non-specifically bound proteins were removed with 10 column volumes of buffer A containing 50 mM imidazole. The hexahistidine-tagged recombinant proteins were then eluted with three column volumes of buffer A containing 250 mM imidazole. The purity and subunit molecular masses of the recombinant ORS and CHS were verified by an SDS-PAGE analysis (Laemmli, 1970), and the protein concentrations were measured by the Bradford method (Bradford, 1976). The native molecular masses of the recombinant proteins were determined by gel filtration chromatography on a 2.5 cm × 75 cm column of Sephacryl S-200 HR resin (GE Healthcare), calibrated with standard proteins.

### Bacterial Expression and Purification of the Recombinant *C. sativa* OAC

The recombinant *C. sativa* OAC was bacterially expressed and purified as a homogeneous protein with a molecular mass of ∼12 kDa, as described previously (Yang et al., 2015).

### Enzyme Assays

The standard reaction mixture consisted of 100 μM starter-CoA, 200 μM malonyl-CoA, 20 μg of the purified recombinant ORS or CHS, and 100 mM potassium phosphate buffer (pH 7.0) in a total volume of 500 μl. The reactions were incubated at 30°C for 30 min. The ORS reactions coupled with OAC were conducted in a similar manner, including 7, 20, or 50 μg of the purified OAC. Afterward, the reaction products were extracted twice with 500 μl of ethyl acetate. The organic layer was evaporated to dryness, dissolved in 100 μl of methanol, and analyzed by HPLC and LC-ESI-MS.

### HPLC and LC-ESI-MS Analyses of the Enzyme Reaction Products

The reaction products were routinely analyzed and quantified by an HPLC system (Tosho, Tokyo, Japan) equipped with a Cosmosil 5C18-MS-II column (4.6 mm × 150 mm, Nacalai Tesque, Tokyo, Japan), as described previously (Taura et al., 2014). Elution was performed with H_2_O and acetonitrile, both containing 0.1% formic acid, at a flow rate of 0.4 ml/min. The gradient programs were as follows. Program A (for the acetyl-CoA primed reactions): 0–5 min, 10% acetonitrile; 5–20 min, 10–25% acetonitrile; 20–40 min, 25% acetonitrile. Program B (for the reactions with starter substrates other than acetyl-CoA): 0–5 min, 10% acetonitrile; 5–20 min, 10–40% acetonitrile; 20–40 min, 40% acetonitrile. The polyketide products were detected by the absorption at 280 nm, and quantified from the peak areas using calibration curves of each standard compound.

The samples were also analyzed by an LC-ESI-MS system (Thermo Fisher Scientific), composed of an Accela 600 HPLC pump, an Accela PDA detector, and an LTQ-Orbitrap-XL ETD Hybrid Ion Trap-Orbitrap Mass Spectrometer, to characterize the products in detail. The column and solvent systems were the same as those used for the HPLC analysis, except that UV spectra were collected with a PDA detector within the range from 200 to 400 nm. The reaction products were ionized in the negative ion mode, with a scan range from *m*/*z* 100 to 500. The LTQ-Orbitrap-XL was operated in the data-dependent parallel detection mode, in which the scan cycles start with a full scan of high-resolution Fourier transformation MS, followed by MS/MS scans in the linear ion trap of the most abundant precursor ions.

The reaction products were identified by using authentic polyketides obtained in our previous studies (Taura et al., 2009; Mori et al., 2013) or from commercial sources, except for the tetraacetic acid lactone standard, which was purified from the transgenic yeast culture as described below.

### Enzyme Kinetics

The enzyme reactions were conducted in a similar manner as the standard assay conditions, using six concentrations (10, 20, 40, 60, 80, and 100 μM) of starter substrates in the presence of 200 μM malonyl-CoA, and the products were quantified by HPLC. The kinetic constants were calculated by fitting the polyketide-forming velocity data at each starter substrate concentration to Hanes-Woolf plots (Hofstee, 1952).

### Production of Polyketides in the Transgenic *P. pastoris* Culture

The ORS coding sequence was amplified with the primers ORS_Fw2 and ORS_Rv2 (Supplementary Table 1). The ORS_Fw2 primer contains a partial Kozak sequence (AAAACA) prior to the translational start ATG codon, for optimal yeast expression (Hamilton et al., 1987). The amplified fragment was subcloned into the pPICZA vector (Thermo Fisher Scientific), predigested with *Eco*RI and *Sal*I, by the In-Fusion HD reagent. The resulting construct was linearized by *Sac*I digestion, and transformed into *P. pastoris* KM71H (Thermo Fisher Scientific) by electroporation. The transgenic *P. pastoris* was selected on YPD agar plates containing 1,000 μg/ml zeocin. The transgenic control strain was prepared in a similar manner, using the empty pPICZA vector.

Protein expression in *P. pastoris* was accomplished essentially as described by Weis et al. (2004). The three minimal media used herein contained 200 mM potassium phosphate buffer (pH 6), 1.34 (w/v)% yeast nitrogen base and 4 × 10^-5^ (w/v)% D-biotin, and differed with respect to the carbon source of 10 g/l of glucose, 1 or 5 (v/v)% of methanol for BMD, BMM2 or BMM10, respectively. A single colony was inoculated into Erlenmyer flasks containing 100 ml BMD, and cultivated at 25°C for 60 h. Then, 100 ml of BMM2 was added to the culture to initiate the induction of ORS gene expression by methanol. BMM10 (20 ml) was fed at 10, 24, 48, 72, 96, and 120 h after the onset of the induction. A 5 ml aliquot of the culture was withdrawn immediately after the addition of BMM2 or BMM10, and then centrifuged to obtain the culture supernatant and the cell pellet. The cells were washed twice with water, and then vigorously vortexed with 50% methanol in the presence of glass beads to prepare the cellular extract. The polyketides in the culture medium and the cellular extract were quantified by HPLC analyses.

### Purification of Tetraacetic Acid Lactone from the Transgenic *P. pastoris*

The transgenic *Pichia* was cultured, and the protein expression was induced as described above. The culture supernatant after a 96 h induction period was extracted twice with an equal volume of ethyl acetate, and the collected organic layer was evaporated. Tetraacetic acid lactone was then purified by preparative HPLC, using a Cosmosil 5C18-MS-II column (10 mm × 250 mm, Nacalai Tesque) eluted with 10% acetonitrile containing 0.1% formic acid, at a flow rate of 5.0 ml/min. Consequently, 1.2 mg of the product was obtained from 1,000 ml culture supernatant. The LC-ESI-MS analysis, performed as described above, identified the obtained product as tetraacetic acid lactone, as its HR-MS, MS/MS, and UV data were identical to those previously reported (Springob et al., 2007).

Tetraacetic acid lactone (6-acetonyl-4-hydroxy-2-pyrone): HR-MS (ESI) Anal. Calcd for [C_8_H_7_O_4_]^-^ *m*/*z* 167.03444 [M-H]^-^, Found 167.03448; MS/MS *m*/*z* 125.1 [C_6_H_5_O_3_]^-^, 123.1 [M-H-CO_2_]^-^; λ_max_ (PDA), 283 nm.

### Expression Analyses of ORS and CHS Genes by Semi-Quantitative RT-PCR

Total RNA was isolated from young leaves, mature leaves, twigs, flowers, and roots of *R. dauricum*, as described above. The first-strand cDNA was then synthesized from 1.0 μg of each RNA sample as described above, except that a random hexamer (Toyobo) was used as the primer. The ORS gene fragment (1,001 bp) was amplified with the primers ORS_3R and ORS_Rv, while the primers CHS_3R and CHS_Rv were used to detect the CHS transcript (873 bp). The 18S rRNA gene (Genbank: AB973224.1, 663 bp), amplified with the primers 18S_Fw and 18S_Rv, was used as a loading control for the agarose gel electrophoresis of PCR products.

### Tissue Distribution of DCA

Methanolic extracts were prepared from young leaves, mature leaves, twigs, flowers, and roots of *R. dauricum*, and the DCA contents were analyzed by HPLC as described previously (Taura et al., 2014).

## Results

### Cloning of cDNAs Encoding ORS and CHS from *R. dauricum*

**FIGURE 2:**
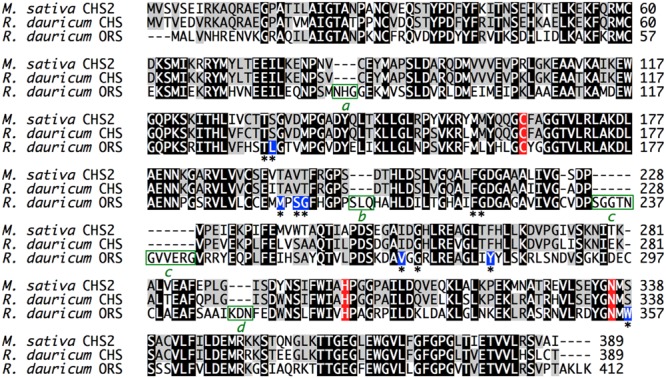
**Multiple amino acid sequence alignment of *M. sativa* CHS2 and *R. dauricum* CHS and ORS.** The catalytic triad residues are colored red, and the active site residues are indicated with asterisks. The varied active site residues in ORS are colored blue. The four inserted peptide sequences *a*–*d* in ORS are highlighted by green boxes.

**FIGURE 3:**
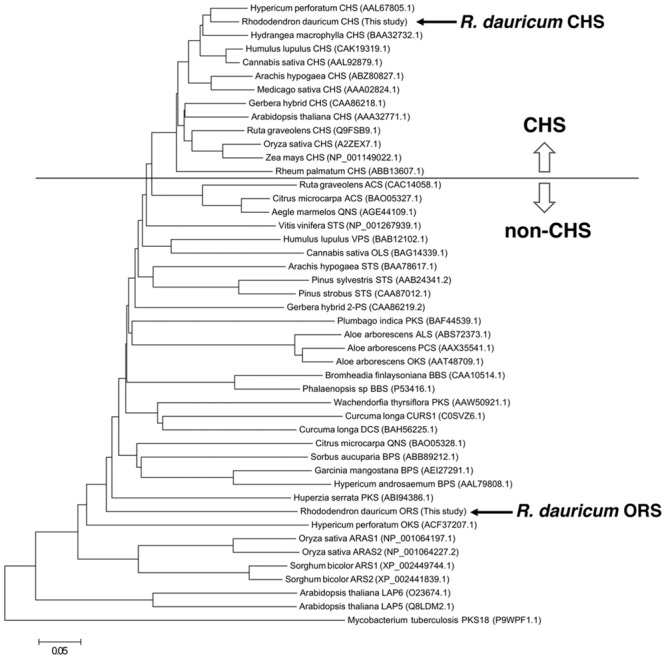
**Phylogenetic tree analysis of plant type III PKSs.** The bacterial type III PKS *Mycobacterium tuberculosis* PKS18 was used as an outgroup. The scale represents 0.05 amino acid substitutions per site. Abbreviations used are: ACS, acridone synthase; ALS, aloesone synthase; ARAS, alkylresorcylic acid synthase; ARS, alkylresorcinol synthase; BBS, bibenzyl synthase; BPS, benzophenone synthase; CHS, chalcone synthase; CURS, curcumin synthase; DCS, diketide-CoA synthase; LAP, Less adhesive pollen; OKS, octaketide synthase; OLS, olivetol synthase; ORS, orcinol synthase; PCS, pentaketide chromone synthase; 2PS, 2-pyrone synthase; QNS, quinolone synthase; STS, stilbene synthase; VPS, valerophenone synthase. The NCBI protein registration numbers are in the parentheses.

The cDNA encoding a novel type III PKS, ORS, was cloned and sequenced together with the CHS cDNA from young leaves of *R. dauricum*, by PCR using degenerate primers and RACE strategy. The CHS cDNA contained a 1,170-bp open reading frame encoding a 389-amino acid polypeptide with a molecular mass of 42,599 Da, and shared very high levels (∼90%) of identity to known CHS sequences. The identity between *R. dauricum* CHS and the structurally characterized *M. sativa* CHS2 (Ferrer et al., 1999) was also significant (∼87.6%; **Figure 2**). In addition, all of the amino acid residues in the CHS active site are conserved in *R. dauricum* CHS (**Table 1**). A phylogenetic tree analysis grouped *R. dauricum* CHS within the CHS clade (**Figure 3**). Based on this sequence information, the CHS cDNA is expected to encode an active CHS in *R. dauricum*.

**Table 1:** Comparison of active site cavity residues of *M. sativa* CHS2 and *R. dauricum* CHS and ORS.

*M. sativa* CHS2	*R. dauricum* CHS	*R. dauricum* ORS
**Initiation pocket**
Phe 215	Phe 215	Phe 218
Ile 254	Ile 254	Val 269
Gly 256	Gly 256	Gly 271
Phe 265	Phe 265	Tyr 280
**Elongation pocket**
Thr 132	Thr 132	Thr 132
Ser 133	Ser 133	Leu 133
Thr 194	Thr 194	Met 194
Val 196	Val 196	Ser 196
Thr 197	Thr 197	Gly 197
Gly 216	Gly 216	Gly 219
Ser 338	Ser 338	Trp 357
**Catalytic triad**
Cys 164	Cys 164	Cys 164
His 303	His 303	His 322
Asn 336	Asn 336	Asn 355

*The residues varied in ORS are underlined.*

In contrast, the ORS cDNA encoded a unique protein. The gene includes a 1,239-bp open reading frame encoding a 412-amino acid polypeptide, with a molecular mass of 45,707 Da. The deduced primary structure of ORS revealed the presence of the conserved the Cys-His-Asn catalytic triad, but had relatively low identities to those of plant PKSs (less than 60%); e.g., ∼54.3% identity to *M. sativa* CHS2 (**Figure 2**). The ORS polypeptide was slightly larger than the other plant PKSs, because of the unique insertions of four peptide sequences *a*–*d*, as shown in **Figure 2**. In addition, as listed in **Table 1**, there were several amino acid substitutions in the CHS active site of ORS (Ferrer et al., 1999): Ile254 and Phe265 in the initiation pocket and Ser133, Thr194, Thr197 and Ser338 in the elongation/cyclization pocket (numbering in *M. sativa* CHS2) were replaced by other amino acids, respectively. These residues have been proposed to regulate the substrate specificity and the catalytic properties of the CHS-related enzymes (Ferrer et al., 1999; Jez et al., 2000; Austin et al., 2004). Furthermore, ORS was grouped with the non-chalcone producing enzymes in the phylogenetic analysis (**Figure 3**). Thus, we considered that ORS could be a novel enzyme derived from CHS.

### Homology Modeling and Structural Characteristics of ORS

**FIGURE 4:**
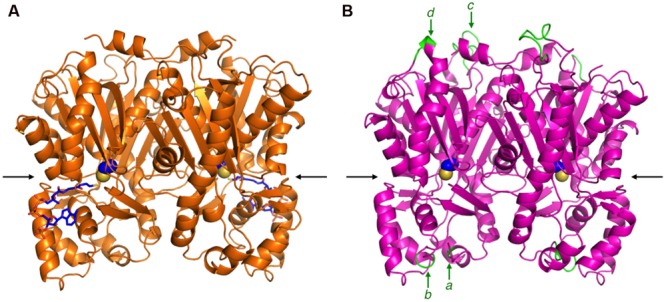
**The overall structures of *M. sativa* CHS2 and *R. dauricum* ORS. (A)** The crystal structure of *M. sativa* CHS2 in complex with CoA (PDB: 1BQ6). The CoA molecule is depicted as blue sticks. **(B)** The model structure of ORS. The active site Cys164 in both enzymes is represented by a CPK model. The arrows indicate active site entrances. The inserted peptide sequences *a*–*d* in ORS are colored green and indicated by arrows.

The primary structure comparison of ORS and CHS revealed several active site residue substitutions and unique peptide insertions. To assess the effects of these sequence changes on the protein structure, a homology model of ORS was constructed with the crystal structure of *M. sativa* CHS2 as a template, wherein the Ramachandran plot calculated that ∼96.7% of the amino acid residues were grouped in the energetically allowed regions. **Figure 4B** shows the overall structure of the ORS model, along with the crystal structure of *M. sativa* CHS2 (**Figure 4A**). Despite the relatively low sequence homology between the two enzymes, ORS adopts almost the same three-dimensional overall fold as that of *M. sativa* CHS2. In addition, all of the inserted peptide sequences in ORS were located on the protein surface, far from the active site cavity (**Figure 4B**), suggesting that these insertions minimally affect the protein folding and the catalytic activity.

**FIGURE 5:**
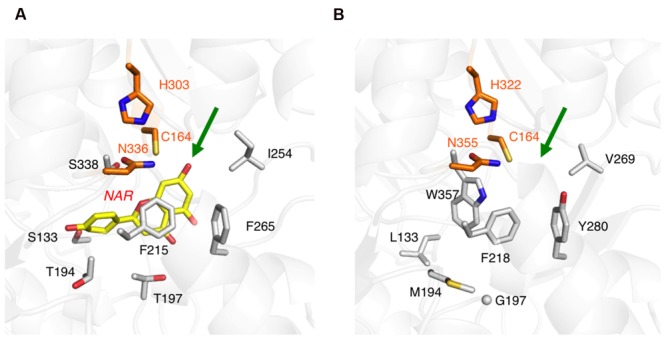
**Close-up views of the enzyme active sites. (A)** *M. sativa* CHS2 in complex with a product analog, naringenin (PDB: 1CGK), colored yellow. **(B)** The model structure of the ORS active site. The green arrows point to the active site entrances. The catalytic triad residues are colored orange. Only selected amino acid residues are depicted, to clearly illustrate the differences between the two enzymes.

However, the amino acid substitutions in the ORS active site apparently reduced the cavity volume (**Figure 5B**), as compared to that of *M. sativa* CHS2 (**Figure 5A**). In particular, the change of Ser338 to Trp, the novel substitution in plant type III PKSs, drastically reduces the cavity volume of ORS to prevent the entry of large starter substrates, such as *p*-coumaroyl-CoA (**Figure 5B**). In addition to the S338W substitution, the small to large F265Y, S133L, and T194M changes also reduced the active site cavity volume of ORS. In contrast, Thr197 is exceptionally changed to the smallest amino acid, Gly. It has been demonstrated that the T197G substitution opens a novel tunnel at the bottom of the active site, allowing it to accept long chain polyketide intermediates, as reported for the octaketide synthase from *Aloe arborescens* (Abe et al., 2005). However, judging from the ORS model, the large side chain of Met194 protrudes toward the G197 position to partially occlude the space created by the T197G substitution (**Figure 5B**). Taken together, the CASTP program estimated the cavity volume of the ORS model to be 465 Å^3^ for each monomer, which is much smaller than that of *M. sativa* CHS2 (1019 Å^3^) that produces naringenin chalcone from *p*-coumaroyl-CoA and three molecules of malonyl-CoA (Ferrer et al., 1999), and slightly larger than that of *G. hybrida* 2-pyrone synthase (298 Å^3^) that synthesizes triacetic acid lactone from acetyl-CoA and two molecules of malonyl-CoA (Jez et al., 2000).

### Bacterial Expression and Characterization of the Recombinant ORS and CHS

**FIGURE 6:**
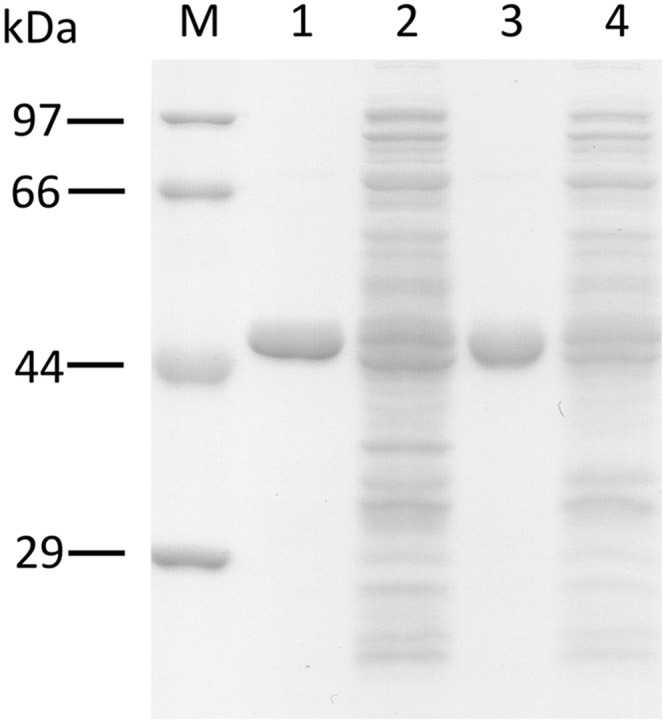
**SDS-PAGE analysis of the purified recombinant PKSs.** M, molecular mass standards, (1) the purified ORS (3 μg), (2) total soluble proteins from *E. coli* expressing ORS, (3) the purified CHS (3 μg), (4) total soluble proteins from *E. coli* expressing CHS.

The recombinant ORS and CHS were bacterially expressed and purified, to evaluate their catalytic functions. The SDS-PAGE analysis demonstrated that the recombinant enzymes were purified as homogeneous proteins with molecular masses of ∼48 and ∼45 kDa (**Figure 6**), which were suitable sizes for hexahistidine-tagged ORS and CHS, respectively. In addition, the gel filtration chromatographies estimated the native molecular masses of ORS and CHS to be ∼101 and ∼94 kDa, respectively, suggesting that the recombinant enzymes are homodimers, as in the cases of known type III PKSs (Austin and Noel, 2003; Abe and Morita, 2010).

The recombinant CHS produced naringenin, derived by the non-enzymatic cyclization of naringenin chalcone (Heller and Hahlbrock, 1980), as the major reaction product from *p*-coumaroyl-CoA and malonyl-CoA, together with bis-noryangonin and coumaroyl triacetic acid lactone, which are common products of *in vitro* CHS reactions (Akiyama et al., 1999) (Supplementary Figure 1). The LC-ESI-MS data for the respective products are summarized in Supplementary Table 2. In addition, the recombinant CHS did not yield tetraketide-derived products such as OSA, but afforded only the triacetic acid lactone from acetyl-CoA and malonyl-CoA (Supplementary Table 2). These results confirmed that *R. dauricum* CHS is a typical CHS, and is not involved in OSA biosynthesis.

**FIGURE 7:**
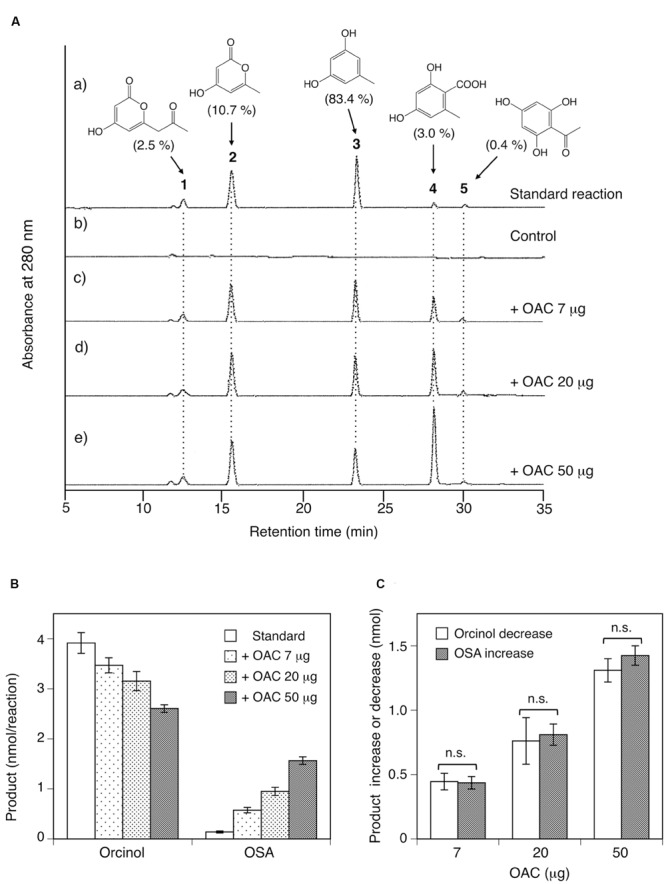
**Analysis of reaction products afforded by the recombinant ORS from acetyl-CoA and malonyl-CoA. (A)** HPLC elution profiles of (a) the standard reaction using 20 μg of ORS, (b) the control reaction with heat-denatured ORS, and (c–e) the reactions using 20 μg (∼0.42 nmol) of ORS, along with 7, 20, or 50 μg (∼0.58, 1.7, and 4.2 nmol) of *C. sativa* OAC, respectively. The percentages of each product under standard assay conditions are shown in the parentheses. Note that each product has different molar extinction coefficients, and thus the peak intensities are not equal to the product percentages in the parentheses. **(B)** Orcinol and OSA product amounts from reactions without or with the indicated amounts of *C. sativa* OAC. Data are means ± SD of triplicate determinations. **(C)** OAC-dependent orcinol decrease and OSA increase, as compared with the product amounts under standard assay conditions. Data are means ± SD of triplicate determinations. n.s., not significant by Student’s *t*-test (*P* > 0.05).

In contrast to CHS, an HPLC analysis confirmed that the recombinant ORS produced five reaction products (products **1–5**) from acetyl-CoA and malonyl-CoA (**Figure 7A**). The LC-ESI-MS analysis of the reaction mixture identified these products as tetraacetic acid lactone (**1**), triacetic acid lactone (**2**), orcinol (**3**), OSA (**4**), and phloroacetophenone (**5**), based on direct comparisons of their retention time, HR-MS, MS/MS, and UV-VIS data to those obtained for authentic samples, as listed in Supplementary Table 3. The ratio of the products under the standard conditions was 2.5% (**1**), 10.7% (**2**), 83.4% (**3**), 3.0% (**4**), and 0.4% (**5**), respectively, suggesting that ORS predominantly produces orcinol (**3**). In the ORS reaction, orcinol is not the decarboxylation product of enzymatically synthesized OSA, since the product ratio between oricinol and OSA was always constant regardless of the incubation period. It should be noted that ORS is the first plant type III PKS that produces aromatic polyketides from an acetate-derived tetraketide (methyl tetra-β-ketide) intermediate. Although ORS contains the T197G substitution that could affect the polyketide size (Abe et al., 2005), we could not detect large reaction products, such as pentaketides, in the ORS reaction mixture.

As shown in **Table 2**, a kinetic analysis of ORS reactions with acetyl-CoA clearly demonstrated that this enzyme synthesized orcinol with a higher *k*_cat_ value than those for the other reaction products. The catalytic efficiency (*k*_cat_/*K*_m_) for the orcinol formation was 2,432 s^-1^M^-1^, which was of the same order of magnitude as those reported for the alkylresorcinol synthases from *Sorghum bicolor* and *Azotobacter vinelandii* (Funa et al., 2006; Cook et al., 2010). In addition, the *k*_cat_/*K*_m_ for the OSA formation (114 s^-1^M^-1^) was relatively low, but was similar to those of the ARASs from rice (Matsuzawa et al., 2010). ORS also synthesized phloroacetophenone via a CHS-like C1-C6 Claisen condensation, but the *k*_cat_/*K*_m_ value (13.6 s^-1^M^-1^) was much lower than those of the CHS enzymes (Jez et al., 2002; Abe et al., 2004). For example, the *R. dauricum* CHS obtained in this study synthesized naringenin from a *p*-coumaroyl-CoA starter with the following kinetic constants: *K*_m_ = 8.15 μM, *k*_cat_ = 0.534 min^-1^, and *k*_cat_/*K*_m_ = 1,093 s^-1^M^-1^. Nevertheless, it was reasonable that ORS produced a small amount of phloroacetophenone, because *R. dauricum* contains 4-*O*-methylphloroacetophenone as a minor constituent (Anetai et al., 1995). With respect to the substrate specificity, ORS produced triketide pyrones with modest catalytic efficiencies when the enzyme was incubated with aliphatic-CoA starters with short side chains, such as butyryl-CoA (C4) and hexanoyl-CoA (C6; **Table 2**), whereas no products were synthesized when *p*-coumaroyl-CoA and aliphatic-CoA starters larger than octanoyl-CoA (C8) were used as the substrates. These results indicated that ORS exclusively accepts starter-CoAs with short aliphatic side chains, probably because of the narrow active site, and primarily preferred acetyl-CoA.

**Table 2:** Steady state kinetic parameters of ORS.

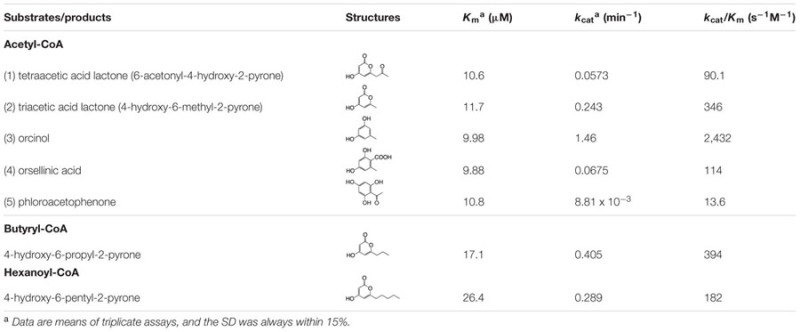

### OAC-Dependent Product Changes of ORS Reactions

ORS exhibited intriguing catalytic properties; however, the major reaction product is not OSA, the precursor of DCA, but orcinol (**Figure 7A**). Thus, we suspected that, as in the case of the olivetolic acid biosynthesis in *C. sativa* (**Figure 1B**), ORS might function as a TKS in the OSA biosynthesis in *R. dauricum*: ORS produces a linear tetraketide intermediate as a substrate for an accessory protein, like OAC, to cyclize it into OSA *in vivo*. To simply assess this possibility, various amounts (7, 20, and 50 μg) of the recombinant OAC protein were added to the standard assay mixture containing 20 μg of ORS, and the product pattern was analyzed by HPLC. As a result, OAC apparently affected the ratio of the orcinol (**3**) and OSA (**4**) products: OAC accelerated the OSA production and decreased the orcinol amount in a dose-dependent manner (**Figure 7**), whereas it did not affect the amounts of the tetraacetic acid lactone (**1**), triacetic acid lactone (**2**), and phloroacetophenone (**5**) products (**Figure 7A**). Interestingly, the quantitative analysis demonstrated that the rate of the orcinol decrease was always equal to that of the OSA increase (**Figures 7B,C**), implying that the OSA synthesis by OAC and the orcinol formation compete for the same linear polyketide intermediate, as discussed below.

### Polyketide Production in the Culture of Transgenic *Pichia pastoris* Harboring the ORS Gene

**FIGURE 8:**
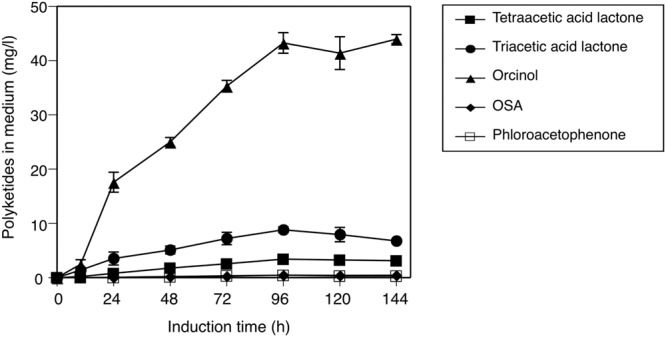
**Secreted production of polyketides by the transgenic *P. pastoris* harboring the ORS gene.** Induction time indicates the period after the onset of ORS expression by feeding media containing methanol. The polyketide data are means ± SD of triplicate determinations by HPLC.

In this study, we also tried to heterologously express ORS in the methylotrophic yeast *P. pastoris*, in order to partially mimic the situation in the plant cells, and to determine whether the catalytic properties of ORS are altered *in vivo*. The liquid culture of transgenic *P. pastoris* secreted all of the expected polyketide metabolites into the culture medium (**Figure 8**), and negligible amounts of products were detected in the cellular extracts throughout the culture period. In contrast, no polyketide products accumulated in the culture of the control *P. pastoris* transformed with an empty vector. Therefore, ORS is functionally expressed in *Pichia* cells, and it synthesized polyketides from endogenous substrates and effectively secreted the products into the medium. The secreted polyketides from the transgenic *Pichia* reached the maximum at 96 h after the onset of protein expression, with product proportions similar to those of the *in vitro* ORS reaction (**Figure 8**). This result suggested that orcinol (or methyl tetra-β-ketide CoA) is the major product of ORS even in an *in vivo* environment.

### Expression and Possible Physiological Functions of ORS and CHS in *R. dauricum*

**FIGURE 9:**
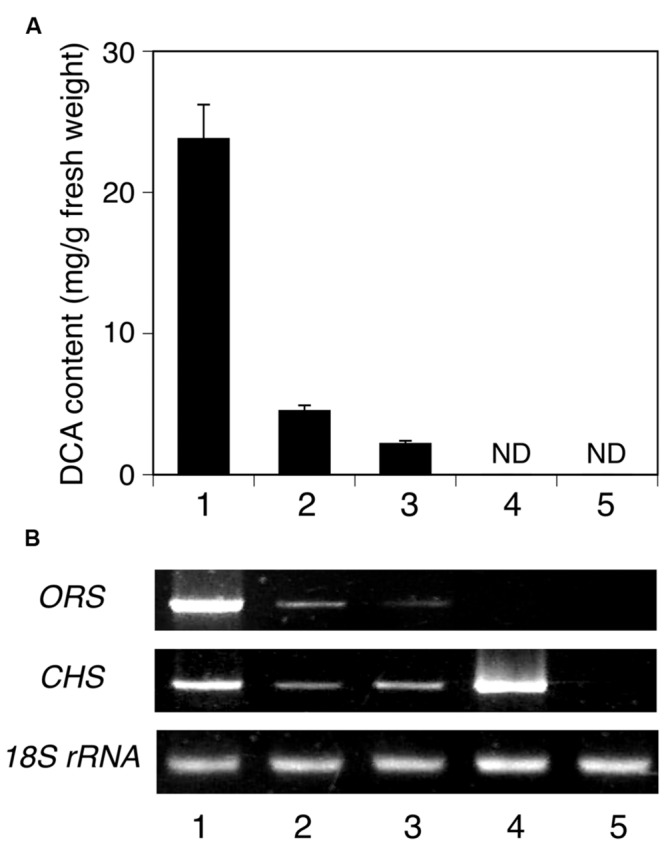
**Analyses of the tissue distributions of DCA and PKS transcripts in *R. dauricum*. (A)** DCA content in various tissues, analyzed by HPLC. Numbers indicate young leaves (1), mature leaves (2), twigs (3), flowers (4), and roots (5). The data are means ± SD of triplicate assays. ND, not detected. **(B)** Semi-quantitative RT-PCR analyses of ORS and CHS gene expression. The 18S rRNA gene fragment was amplified as a housekeeping gene. Numbers indicate the same tissues as shown in **(A)**.

The tissue-specific expression of ORS and CHS in *R. dauricum* plants was analyzed by semi-quantitative RT-PCR experiments, using gene-specific primers. As shown in **Figure 9B**, the ORS gene is clearly expressed at the highest level in young leaves, and in lesser amounts in mature leaves and twigs. This distribution pattern agreed well with the DCA content in each tissue (**Figure 9A**). In contrast, the CHS gene was mostly expressed in flowers, followed by young leaves. Thus, the CHS herein obtained seems to participate in the flavonoid biosynthesis in flowers and young leaves, as *R. dauricum* produces various flavonoids in these tissues (Qiang et al., 2011).

## Discussion

In the present study, we cloned the cDNA encoding ORS, a novel plant type III PKS, from young leaves of *R. dauricum*. Notably, as compared with CHS, the primary structure of ORS contained simultaneous amino acid changes in the CHS’s conserved active site residues, although it conserved the Cys-His-Asn catalytic triad commonly found in the type III PKSs. *In vitro* enzyme assays revealed that the recombinant ORS did not accept *p*-coumaroyl-CoA, the typical starter molecule for plant type III PKSs, but preferred acetyl-CoA as the starter to produce five reaction products via two to three condensations with malonyl-CoA. Multiple product formation from small starter molecules has also been reported for several plant PKSs (Abe and Morita, 2010). For example, *Hypericum perforatum* PKS2 catalyzed the condensation of acetyl-CoA with two to seven malonyl-CoAs to yield cyclic tri- to octa-ketide products (Karppinen et al., 2008). Likewise, a PKS from *Plumbago indica* could synthesize tri- to hexa-ketide products via sequential condensations of malonyl-CoA to acetyl-CoA (Springob et al., 2007). However, it should be noted that the ORS identified herein is the first type III PKS producing aromatic tetraketides; namely, orcinol, OSA, and phloroacetophenone. In other words, these simple phenols were identified for the first time as plant PKS products. The catalytic properties of ORS were thus novel and remarkable. However, ORS was not expected to synthesize orcinol as the major reaction product, because *R. dauricum* does not contain neutral meroterpenoids such as confluentin and grifolin, the decarboxylated forms of DCA and grifolic acid (Taura et al., 2014). To obtain some clues to solve this puzzle between the ORS products and the *in planta* metabolites, we conducted ORS reactions including various amounts of OAC, the only plant polyketide cyclase known to produce olivetolic acid (Gagne et al., 2012; Yang et al., 2016). As a result, quite interestingly, OAC dose-dependently increased the OSA production and simultaneously decreased the orcinol formation, whereas it did not affect the amounts of the other side products. Therefore, as in the case of the olivetolic acid biosynthesis in *C. sativa* (**Figure 1B**), OAC produced a resorcylic acid ring system in collaboration with ORS.

**FIGURE 10:**
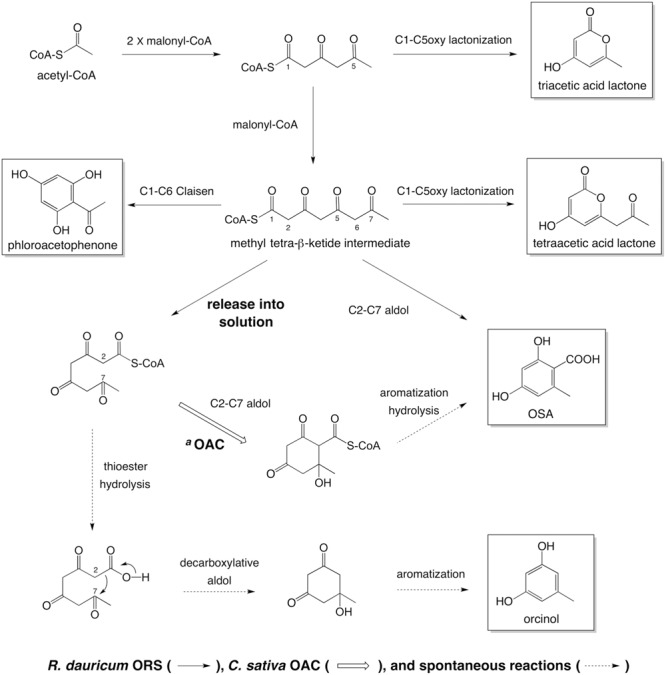
**Summary of the reactions catalyzed by ORS, from acetyl-CoA as a starter substrate.** ^a^In the presence of *C. sativa* OAC, methyl tetra-β-ketide CoA, released from the ORS active site, undergoes aldol condensation to form a C2–C7 linkage. The following aromatization and thioester hydrolysis could take place in solution to yield OSA. In the absence of OAC, the tetraketide CoA would be spontaneously cyclized into orcinol.

The simplest scenario explaining the OAC-dependent product change is illustrated, based on the reported biochemical properties of OAC (Gagne et al., 2012; Yang et al., 2016) (**Figure 10**). Like *C. sativa* TKS (Taura et al., 2009), ORS produces and releases considerable amounts of a tetraketide (methyl tetra-β-ketide CoA), perhaps as the “real” major product. In the absence of OAC, the tetraketide is non-enzymatically cyclized to orcinol by decarboxylative aldol condensation, via a reaction scheme in the order of (1) thioester hydrolysis, (2) aldol condensation accompanied by decarboxylation, and (3) aromatization, as proposed for various alkylresorcinols and stilbene biosynthetic reactions (Austin et al., 2004; Funa et al., 2006; Taura et al., 2009; Cook et al., 2010). In contrast, OAC accepts the non-physiological substrate, methyl tetra-β-ketide CoA, to form the C2–C7 linkage before the thioester cleavage (**Figure 10**), as in the case of olivetolic acid biosynthesis. Since OAC lacks aromatase and thioesterase domains (Yang et al., 2016), the following aromatization and thioester hydrolysis would take place in solution, to form OSA (**Figure 10**). The OAC active site contains a hydrophobic pentyl-binding pocket that is important for the recognition and binding of the physiological substrate, pentyl tetra-β-ketide CoA (Yang et al., 2016). Thus, methyl tetra-β-ketide CoA, supplied by ORS, might not be a preferable substrate for OAC, and the OAC-dependent aldol condensation competes with the spontaneous orcinol formation even when an excess amount of OAC is present, as shown in **Figure 7**. Nevertheless, this study provided the first evidence that OAC can partly accept methyl tetra-β-ketide CoA as a substrate, suggesting the possibility that the rational modification of the OAC active site, especially the pentyl-binding pocket, based on the crystal structure (Yang et al., 2016) could create mutant OACs with novel substrate specificities. In contrast to orcinol, the production of tetraacetic acid lactone and phloroacetophenone was not affected by adding OAC (**Figure 7A**). These tetraketide-derived products are likely to be synthesized in the active site of ORS, rather than outside the enzyme. It is also notable that, unlike *C. sativa* TKS, ORS could synthesize resorcylic acid OSA in the absence of a cyclase, with a catalytic efficiency similar to those of the *O. sativa* ARASs. These results suggested the multifunctional nature of the ORS active site, which can catalyze both the C1–C6 Claisen and C2–C7 aldol reactions, in addition to the C1–C5oxy lactonization of the same methyl tetra-β-ketide intermediate (**Figure 10**).

We also confirmed that the ORS gene is predominantly expressed in young leaves, the DCA-producing tissue of *R. dauricum* (Taura et al., 2014). This tissue distribution pattern of ORS transcripts, along with the substrate specificity and the catalytic properties of the recombinant enzyme, supports the possibility that ORS is the PKS involved in OSA biosynthesis, in combination with an unidentified cyclase, in plants. To demonstrate the expression of the OSA-producing cyclase, we prepared crude protein extracts from young leaves of *R. dauricum*, and included them in the ORS assays. However, the corresponding cyclase activity has not been detected yet, probably because of an insufficient expression level or the *in vitro* instability of the cyclase. Accordingly, an RNAseq analysis of *R. dauricum* young leaves is now in progress in our laboratories to directly explore the candidate genes for the OSA-producing cyclase with sequence and/or structural similarities to known enzymes, including the *C. sativa* OAC and *Streptomyces* type II polyketide cyclases (Shen and Hutchinson, 1993, 1996; Torkkell et al., 2000; Sultana et al., 2004; Thompson et al., 2004; Ames et al., 2008; Yang et al., 2016). In addition, ORS showed the quite interesting catalytic properties of producing multiple tetraketide-derived simple phenols, as a novel catalytic repertoire for a plant type III PKS. The amino acid substitutions observed in the ORS active site presumably provide these remarkable catalytic functions. ORS is thus an interesting enzyme to study the complex catalytic potential hidden in a structurally simple type III PKS, by means of crystal structure analysis and site-directed mutagenesis. Results from these continuing studies will be communicated in due course.

## Author Contributions

FT and HM conceived and designed this study; FT, MI, EY, and HS performed the experiments; HT and HK provided assistance in molecular cloning and various computational analyses; SM, YA, and FK supervised the project; and FT, MI, FK, and HM wrote the manuscript.

## Conflict of Interest Statement

The authors declare that the research was conducted in the absence of any commercial or financial relationships that could be construed as a potential conflict of interest.

## Footnotes

1 http://www.mbio.ncsu.edu/BioEdit/bioedit.html

2 http://www.pymol.org
