# Supplementary material for: A Novel Class of Plant Type III Polyketide Synthase Involved in Orsellinic Acid Biosynthesis from Rhododendron dauricum
Source: Front Plant Sci. 2016 Sep 27;7:1452. doi: 10.3389/fpls.2016.01452 (PMC5037138; doi:10.3389/fpls.2016.01452)
Supplement: Supplementary file 1 [file Table_1.DOCX]

Supplementary Material

A novel class of plant type III polyketide synthase involved in orsellinic acid biosynthesis from *Rhododendron dauricum*

**Futoshi Taura^*^, Miu Iijima, Eriko Yamanaka, Hironobu Takahashi, Hiromichi Kenmoku, Haruna Saeki, Satoshi Morimoto, Yoshinori Asakawa, Fumiya Kurosaki, Hiroyuki Morita^*^**

*** Correspondence:**

Futoshi Taura

Graduate School of Medicine and Pharmaceutical Sciences for Research, University of Toyama, 2630 Sugitani, Toyama, 930-0194, Japan. Email taura@pha.u-toyama.ac.jp

Hiroyuki Morita

Institute of Natural Medicine, University of Toyama, 2630 Sugitani, Toyama, 930-0194, Japan. Email morita@inm.u-toyama.ac.jp

**Supplementary Figure 1.** HPLC elution profile of the products synthesized by the recombinant *R. dauricum* CHS, from *p*-coumaroyl-CoA and malonyl-CoA. (A) The standard reaction using 20 μg of CHS. The reaction products are coumaroyl triacetic acid lactone (1), bis-noryangonin (2), and naringenin (3). (B) Control reaction with heat-denatured CHS.

**Supplementary Table 1.** Primers and PCR conditions used in this study.

| Purposes | Primers | Sequences | PCR conditions |
| --- | --- | --- | --- |
| Degenerate PCR | PKS_Fw *^a^*  PKS_Rv *^a^* | TAYCCIGAYTWYTAYTT  CCCCAYTCYTYDATIGC | 30 cycles of (94 ˚C 1 min, 50˚C 1 min, 72 ˚C 1 min) |
| ORS 3’RACE  ORS first 5’RACE  ORS nested 5’RACE  CHS 3’RACE  CHS first 5’RACE  CHS nested 5’RACE | ORS_3R  ORS_5R1  ORS_5R2  CHS_3R  CHS_5R1  CHS_5R | TGAATCATGGTGGGGAGAAG  (AP for the counter primer)  GTCCAGAACATCAAGAGATG  (dT_17_AP for the counter primer)  TCGTTCACGTGCATGTACCT  (AP for the counter primer)  GTGGAAGTTCCCAAATTGGG  (AP for the counter primer)  AACTTCTCTTTGAGCTCTGC  (dT_17_AP for the counter primer)  CCCAGTTTAGGGACCTCAAC  (AP for the counter primer) | 30 cycles of (94 ˚C 1 min, 60˚C 1 min, 72 ˚C 1 min)  5 cycles of (94 ˚C 1 min, 50 ˚C 1 min, 72 ˚C 1 min); 30 cycles of (94 ˚C 1 min, 60 ˚C 1 min, 72 ˚C 1 min)  30 cycles of (94 ˚C 1 min, 60 ˚C 1 min, 72 ˚C 1 min)  ditto  5 cycles of (94 ˚C 1 min, 50 ˚C 1 min, 72 ˚C 1 min);  30 cycles of (94 ˚C 1 min, 60 ˚C 1 min, 72 ˚C 1 min)  30 cycles of (94 ˚C 1 min, 60 ˚C 1 min, 72 ˚C 1 min) |
| ORS full length PCR | ORS_Fw | CGGGATCCATGGCTTTGGTGAACCACA *^b^* | 30 cycles of (98˚C 10 sec, 60˚C 5 sec, 72˚C 1 min) |
|  | ORS_Rv | CCGGTCGACCTATTTAAGTTTGGCCGTAGG *^c^* |  |
| CHS full length PCR | CHS_Fw | CGGGATCCATGGTCACCGTCGAGGATGT *^b^* | ditto |
| ORS yeast expression  ORS RT-PCR  CHS RT-PCR  18S rRNA RT-PCR | CHS_Rv  ORS_Fw2  ORS_Rv2  18S_Fw  18S_Rv | CCGGTCGACTCAAGTGCACAAACTGTGCA *^c^*  CGGAATTCAAAACAATGGCTTTGGTGAACCACA *^d^*  CCGGTCGACTTTAAGTTTGGCCGTAGG *^c^*  ORS_3R and ORS_Rv  CHS_3R and CHS_Rv  TTCTTGGATTTATGAAAGAC  AAGACCAACAATTGCAATGA | ditto  30 cycles of (94 ˚C 1 min, 60 ˚C 1 min, 72 ˚C 1 min)  ditto  25 cycles of (94 ˚C 1 min, 60 ˚C 1 min, 72 ˚C 1 min) |
| First 5’RACE | dT_17_AP | GACTCGTCTAGAGGATCCCG(T)_17_ |  |
| Nested 5’RACE | AP | GACTCGTCTAGAGGATCCCG |  |

*^a^* PKS_Fw and PKS_Rv were designed from the conserved peptide sequences YPD(Y/F)YF and AIKEWG, respectively. *^b^ Bam*HI, *^c^ Sal*I, and *^d^ Eco*RI sites are underlined. The partial Kozak sequence in ORS_Fw2 is double-underlined.

**Supplementary Table 2.** LC-ESI-MS analyses of the reaction products synthesized by *R. dauricum* CHS from acetyl-CoA or *p*-coumaroyl-CoA.

| **Starter-CoA**  products | **Product structures** | **Rt**  (min) | **Gradient *^a^*** | **HR-ESI-MS**  (*m*/*z*) | **MS/MS *^b^***  (*m*/*z*) | ***λ*_max_**  (nm) |
| --- | --- | --- | --- | --- | --- | --- |
| **Acetyl-CoA**  triacetic acid lactone  (4-hydroxy-6-methyl-2-pyrone)  ***p*-Coumaroyl-CoA**   1. 1) coumaroyl triacetic acid lactone 2. 2) bis-noryangonin 3. 3) naringenin |  | 15.8  12.8  17.4  26.8 | A  B  B  B | 125.02413 [M-H]^-^  (calc. for C_6_H_5_O_3_^-^, 125.02387)  271.06046 [M-H]^-^  (calc. for C_15_H_11_O_5_^-^, 271.06065)  229.05000 [M-H]^-^  (calc. for C_13_H_9_O_4_^-^, 229.05009)  271.06054 [M-H]^-^  (calc. for C_15_H_11_O_5_^-^, 271.06065) | 81.1 [M-H-CO_2_]^-^  125.1 [C_6_H_5_O_3_]^-^  227.1 [M-H-CO_2_]^-^  185.1 [M-H-CO_2_]^-^  ND | 283  333  363  289 |
|  |  |  |  |  |  |  |

 *^a^* Solvent systems A and B are described in Materials and Methods. *^b^* Precursor ions are the [M-H]^-^ ions in HR-ESI-MS. Note that the [C_6_H_5_O_3_]^-^ ion corresponds to an α-pyrone core structure, and [M-H-CO_2_]^-^ ions are generated from the decarboxylation of α-pyrone compounds. ND, not determined.

**Supplementary Table 3.** LC-ESI-MS analyses of the reaction products synthesized by ORS from different starter substrates.

| **Starter-CoA**  products | **Product structures** | **Rt**  (min) | **Gradient *^a^*** | **HR-ESI-MS**  (*m*/*z*) | **MS/MS *^b^***  (*m*/*z*) | ***λ*_max_**  (nm) |
| --- | --- | --- | --- | --- | --- | --- |
| **Acetyl-CoA**   1. 1) tetraacetic acid lactone 2. (6-acetonyl-4-hydroxy-2-pyrone)      1. 2) triacetic acid lactone 2. (4-hydroxy-6-methyl-2-pyrone) 3. 3) orcinol 4. 4) orsellinic acid 5. 5) phloroacetophenone   **Butyryl-CoA**  4-hydroxy-6-propyl-2-pyrone  **Hexanoyl-CoA**  4-hydroxy-6-pentyl-2-pyrone |  | 12.6  15.8  23.2  27.9  30.2  12.3  28.3 | A  A  A  A  A    B  B | 167.03474 [M-H]^-^ (calc. for C_8_H_7_O_4_^-^, 167.03444)  125.02420 [M-H]^-^  (calc. for C_6_H_5_O_3_^-^, 125.02387)  123.04490 [M-H]^-^  (calc. for C_7_H_7_O_2_^-^, 123.04461)  167.03400 [M-H]^-^  (calc. for C_8_H_7_O_4_^-^, 167.03444)  167.03441 [M-H]^-^  (calc. for C_8_H_7_O_4_^-^, 167.03444)  153.05544 [M-H]^-^  (calc. for C_8_H_9_O_3_^-^, 153.05517)  181.08647 [M-H]^-^ | 125.1 [C_6_H_5_O_3_]^-^  123.1 [M-H-CO_2_]^-^  81.1 [M-H-CO_2_]^-^  ND  123.0 [M-H-CO_2_]^-^  ND  109.1 [M-H-CO_2_]^-^  137.1 [M-H-CO_2_]^-^ | 283  284  271  261, 297  285  285  285 |
|  |  |  |  | (calc. for C_10_H_13_O_3_^-^, 181.08647) |  |  |
|  |  |  |  |  |  |  |

*^a^* Solvent systems A and B are described in Materials and Methods. *^b^* Precursor ions are the [M-H]^-^ ions in HR-ESI-MS. Note that the [C_6_H_5_O_3_]^-^ ion corresponds to an α-pyrone core structure, and [M-H-CO_2_]^-^ ions are generated from the decarboxylation of α-pyrone and resorcylic acid compounds. ND, not determined.
